# Compulsory Citizenship Behavior and Its Outcomes: Two Mediation Models

**DOI:** 10.3389/fpsyg.2022.766952

**Published:** 2022-02-04

**Authors:** Huai-Liang Liang, Tsung-Kai Yeh, Chia-Hsuan Wang

**Affiliations:** ^1^College of Management, Dayeh University, Changhua, Taiwan; ^2^Department of Management, Air Force Institute of Technology, Kaohsiung City, Taiwan

**Keywords:** compulsory citizenship behavior, emotional exhaustion, deviant behavior, false behavior, impression management

## Abstract

Employees view compulsory citizenship behavior (CCB) as concessionary behavior they undertake because of pressure exerted by their organizations. This study applies affective events theory to CCB-workplace deviance relationships, and impression management theory to CCB-facades of conformity relationships, to posit that employee emotional exhaustion is an essential mediating factor that effectively explains how CCB contributes to workplace deviance and facades of conformity. This study utilizes two mediation models to investigate whether employees’ CCBs are positively related to their work deviance and false behavior, and how emotional exhaustion mediates those relationships. Two-wave data collected from 655 valid participants (480 males, 175 females; average age of 30.1 years) in a public sector bank and a large private bank in Taiwan supported our hypotheses. We conducted surveys with volunteer employees that included CCB, emotional exhaustion, facades of conformity, and work deviance. The results of this study uncovered statistically significant relationships between CCB and work deviance and between CCB and facades of conformity and revealed that emotional exhaustion significantly mediated these relationships. Implications and directions for future study are discussed.

## Introduction

Employees who voluntarily spend extra time at their organizations can produce benefits such as increased work input and improved work performance (Loi et al., 2020). Organ (1988) defined such behavior as organizational citizenship behavior (OCB), in which employees helped and voluntarily contributed to others or the organizations they served. Generally, company management expects employees to perform high levels of OCB in the workplace (Zhao et al., 2014). OCB should in current organization of work more than before because companies face increase competitive market tensions. Therefore, organizations intend to require employees to engage in involuntary and extra-role activities that are valuable to the organization. Although OCB involves acts of personal intention that one can distinguish from employees’ work roles, the employees are more likely to engage in compulsory behavior related to their organizations, and act against their personal intentions, when pressured by others (Bolino et al., 2015). Vigoda-Gadot (2006) defined this type of OCB as compulsory citizenship behavior (CCB), which is a dark side of OCB in organizations and includes the destructive effects of non-voluntary OCB due to the pressure exerted on them by their organizations. CCB is a unique form of citizenship behavior that is less voluntary, where the organization forces its employees to exert extra effort (Vigoda-Gadot, 2006, 2007). In such situations, employees may participate in activities that they were reluctant to join. Indeed, as an unconventional and enforced requirement, the influence of CCBs may negatively affect, and even harm, an organization’s performance, and lead to negative employee behavior (Loi et al., 2020). Therefore, this study focused on whether employees with CCBs violated organizational regulations or policies and performed false behaviors.

Prior research found that CCB is a serious condition, and a frequent issue within Chinese organizations (Lin et al., 2019). Traditional Chinese cultural values influence Chinese employees’ thoughts, causing employees to desire the approval of the organization’s core authorities (He et al., 2019). This increasing stress from employment relationships is a fundamental force that gives Chinese employees no choice but to perform more citizenship behaviors. Prior studies have shown that CCB led to negative employee behaviors such as moral disengagement and employee silence (He et al., 2019), citizenship pressure (Lin et al., 2019), and emotional exhaustion (He et al., 2019). Therefore, we focused on the dark and destructive side of CCB as our research subject, in order to reveal the potential negative consequences of CCB.

This research draws on affective events theory (AET; Weiss and Cropanzano, 1996) that all events that occur in the workplace affect employees’ psychological and emotional reactions. Employees determine the responses of other employees, depending on the organization’s treatment of them (Tripp et al., 2007). Employees are likely to violate organizational regulations if they experience an unpleasant workplace atmosphere. Specifically, employees’ negative feelings about their workplace result in deviant behavior (Mo and Shi, 2017). Employee workplace deviance refers to behavior that violates organizational regulations and policies, or even disturbs organizational order (Peng et al., 2016). Organizations would then need to expend considerable resources to mitigate the losses caused by that workplace deviance. Therefore, we posit that, although employees’ OCB can improve operational processes and organizational performance effectively, the negative feelings and experiences that employees experience while performing OCB (e.g., being forced to engage in citizenship behavior by their organization or supervisors) can potentially lead to workplace deviance.

According to Leary and Kowalski (1990), OCB display is a strategy for impression management. Employees carry out impression management to improve their work processes and organizational performance, although they sometimes implement impression management merely out of self-interest. Loi et al. (2020) advised organizations to recognize the risks and possible consequences of their employees’ voluntary behavior, because employees may display that behavior for impression management rather than altruism. However, CCB stems from the pressure that organizations exert, and describes employees’ reluctance to participate in activities that they do not wish to perform (Vigoda-Gadot, 2006). This implies that employees must conceal their true sentiments in order to comply with their organization’s values and to satisfy its organizational requirements. When employees are forced to perform extra-role behavior, they pretend to conform their thoughts, and suppress their values, by employing an impression management strategy. Employees who reluctantly exhibit extra-role behavior later engage in false behavior to meet organizational requirements. Hewlin (2003, 2009) defined the facade of conformity as the false representation of employees, in which they withhold their values by pretending to embrace their supervisors’ core values. Therefore, we argue that CCB might be a contributing factor when employees engage in facades of conformity and suppress their personal values in order to pander to the values of their supervisors.

Vigoda-Gadot (2006) argued that the antecedent of CCB was the pressure exerted by organizations. In addition, external pressure caused by market competition prompted organizations to enhance their performances in order to maintain their competitiveness (Vigoda-Gadot, 2007). Therefore, employees were likely to experience emotional responses due to the role expansion caused by CCB. Nevertheless, no relevant studies have specified whether employees have had emotional responses after experiencing CCB (He et al., 2019; Liu et al., 2019). Proost et al. (2012) regarded emotional exhaustion as a negative affect, the core component of burnout, and viewed the lack of energy as a sign that that the individual’s emotional resources had been exhausted. Therefore, this study examined how emotional exhaustion plays a mediating role in the relationship between CCB and its consequences (e.g., workplace deviance and facades of conformity). The emotional exhaustion caused by their non-voluntary extra effort at work may cause employees to engage in behavior that violates organizational regulations and suppress their personal values while pandering to those of their supervisors. This study applies AET to CCB-workplace deviance relationships, and impression management theory to CCB-facades of conformity relationships, to posit that employee emotional exhaustion is an essential mediating factor that effectively explains how CCB contributes to workplace deviance and facades of conformity.

In brief, this study has the following three purposes: First, according to both AET and impression management theory, workplace deviance and facades of conformity transpire when companies pressure employees to perform OCB in order to maintain supervisors’ interest. Hence, we explored the influence of CCB on workplace deviance and facades of conformity. Second, according to Vigoda-Gadot (2006), CCB is a type of citizenship behavior that is inconsistent with personal intentions. Due to their false behavior, employees tend to exhibit negative signs such as psychological contradictions, depression, and emotional reactions. Therefore, we investigated how emotional exhaustion mediated the influence of CCB on workplace deviance and facades of conformity. Third, because every event that occurs in the workplace affects employees’ psychological and emotional reactions, as well as their subsequent behavior, we examined whether CCB resulted in negative emotional reactions (e.g., emotional exhaustion) that affected workplace deviance and facades of conformity.

## Literature Review and Hypotheses

### Compulsory Citizenship Behavior and Workplace Deviance

Employees were more likely to perform voluntary acts if they believed that they might receive corresponding rewards; conversely, they reduced their voluntary behavior when organizations reacted negatively to that behavior (Loi et al., 2020). AET emphasizes the processes behind employee emotional reactions in the workplace and focuses entirely on the processes of individual judgment (Weiss and Cropanzano, 1996). Thus, individuals who experienced negative workplace events produced negative emotional responses such as emotional exhaustion, resulting in poorer behavior in the workplace. When employees believed that OCB was beneficial to their personal interests, their motivation to engage in positive emotions increased, and they displayed positive behavior in order to enhance their images with their colleagues.

However, if employees’ voluntary behavior did not result in personal benefits, negative results might follow (Loi et al., 2020). Bolino et al. (2015) revealed that employees become angry and dissatisfied when they were forced to perform extra work or work long hours. In addition to OCB, employees might also exhibit workplace deviance due to certain other factors (Loi et al., 2020). Employees expected to receive special treatment after contributing to their organizations; thus, they might produce workplace deviance when the treatment they received was inconsistent with their expectations. Furthermore, Peng et al. (2016) indicated that employees tended to evaluate whether their workplace treated them fairly. If they believed that they were not treated fairly, they tended to engage in negative behavior. As such, we posit that employees engage in CCB when they expend special effort for their organizations. They might display further workplace deviance if they did not receive corresponding rewards. Accordingly, we propose Hypothesis 1, as follows:

*Hypothesis 1: CCB positively relates to workplace deviance.*

### Compulsory Citizenship Behavior and Facades of Conformity

When employees join an organization, they begin to adapt to the organization’s values. During this socialization, employees adjust to the organizational environment by identifying the types of behavior that the organization accepts. However, some employees tend to suppress their personal values and pretend to agree with the organization’s values, even if the two contradict each other. Hewlin (2003) defined such behavior as facades of conformity, i.e., employees pretending to embrace organizational values. Such false external behavior could encompass specific expressions, gestures, clothing styles, and other signs of consent. It could be a type of learned or even planned behavior (Hewlin, 2003). Façade of conformity can lead to damages for organizations and employees, such as low work engagement (Hewlin et al., 2017), employee voice and job satisfaction (Chou et al., 2020). For instance, employees’ creating facades of conformity to convey the appearance of embracing ideas and values that are dissimilar to their own is likely to high levels of turnover intention among followers (Hewlin, 2009), inauthentic self-presentation, and low organizational learning (Hewlin et al., 2017).

Vigoda-Gadot (2006) asserted that OCB was a type of behavior that was unrewarded in the workplace, where organizations expected employees to voluntarily spend extra time and effort on their work. Moreover, although CCB is false citizenship behavior that employees engage in due to organizational pressure, it remains a type of OCB, and it can improve operational processes and organizational performance. Hewlin (2003) proposed that employees engaging in facades of conformity concealed their true motives through positive external behavior. They enhanced their interpersonal effectiveness in interactions with their colleagues or supervisors by using their personal influence in appropriate contexts. Accordingly, we propose that, after employees constitute motives for conducting impression management, they pander to the expectations of their supervisors and organizations in order to spend additional time on extra-role behavior. Thus, CCB encourages facades of conformity. This leads to our Hypothesis 2, as follows:

*Hypothesis 2: CCB positively relates to facades of conformity*.

### Compulsory Citizenship Behavior and Emotional Exhaustion

According to Maslach and Jackson (1981), employees experienced emotional exhaustion when the emotional resource consumption necessary to complete their work requirements exceeded their emotional resources. Emotional exhaustion was originally considered a measure of job burnout, but it was also effective at explaining the emotional reactions of employees in the workplace. Therefore, the relevant research has examined emotional exhaustion and job burnout separately (Hewlin, 2009). Peng et al. (2016) indicated that employees tended to generate negative emotions and psychological reactions when they failed to manage their stress, and that emotional exhaustion was the most common emotional reaction caused by excessive pressures in the workplace. Emotional exhaustion might have occurred when employees encountered role conflict, role confusion, excessive workload, or work stress (Mo and Shi, 2017). Vigoda-Gadot (2007) revealed that employees were more likely to experience emotional exhaustion and increased work pressure when compelled to engage in extra-role behavior by their organizations.

Continually complying with organizational requirements negatively influenced employees’ physical and psychological health (Whiteside and Barclay, 2013). In such situations, employees constantly consumed their emotional and cognitive resources while attempting to meet organizational requirements; emotional exhaustion then occurred when the consumption exceeded the total resources the employees possessed. Peltokorpi (2019) suggested that, if employees exerted additional effort to address the stress imposed by organizations, they would consume their cognitive resources and experience emotional exhaustion. Lin et al. (2019) argued that continual citizenship behavior prevented employees from completing their tasks while also consuming their resources, eventually resulting in emotional exhaustion. Thus, we posit that employees experience stress because CCB continually consumes their emotional resources and leads to further emotional exhaustion. Accordingly, we establish Hypothesis 3 as follows:

*Hypothesis 3: CCB positively relates to emotional exhaustion*.

### The Mediating Effect of Emotional Exhaustion

AET emphasizes the processes behind employee affective responses in the workplace and focuses entirely on the processes of individual judgment (Weiss and Cropanzano, 1996). Workplace events may produce emotional reactions among employees, leading to attitudinal and behavioral consequences at work. Therefore, every event that occurs in the workplace affects employees’ emotional responses, and their emotional experiences directly influence their behavior. CCB consumes employees’ personal and social resources and induces further emotional exhaustion. Emotional exhaustion tends to occur when other sources cannot supplement the consumption of emotional energy required to satisfy job requirements. In order to reduce the consumption of their personal resources, employees would have to become less committed to their organizations or less engaged in their work. Peltokorpi (2019) argued that emotional exhaustion was the result of workplace stress, and that employees would display other behaviors in response to reduced pressure. Specifically, the emotional exhaustion that employees experience in the workplace might trigger workplace deviance (Peng et al., 2016). According to AET (Weiss and Cropanzano, 1996), when employees encountered negative events caused by CCB, their negative emotional responses or exhaustion triggered workplace deviance. Hence, we propose Hypothesis 4, as follows:

*Hypothesis 4: Emotional exhaustion mediates the relationship between CCB and workplace deviance*.

From an impression management perspective (Liu et al., 2019), self-presentation is the procedure by which people employ impression tactics to influence others’ perceptions of them. Employees experiencing CCB may create facades of conformity by suppressing their individual values in order to positively enhance their ingratiation, whereby they seek to be seen as likable, and to avoid being isolated in the organization. Therefore, employees may create new strategies to meet their needs, such as adopting facades of conformity to meet their employers’ needs (Hewlin, 2009). Given changes in the workplace, organizations expect employees to devote more time and effort toward citizenship behavior so that they can maintain organizational competitiveness. According to AET (Weiss and Cropanzano, 1996), workplace events produce emotional responses and result in certain behaviors. When employees experience exhaustion, they tend to exhibit more false behavior with regard to organizational requirements in order to avoid losing their values in the organization. Liang (2017) revealed that employees would comply with organizational requirements in order to maintain their job security and prevent negative results. Thus, with reference to AET, we propose that employees experience emotional exhaustion when encountering the negative events caused by CCB, which increases their utilization of facades of conformity. Based on the preceding discussion, we propose Hypothesis 5, as follows:

*Hypothesis 5: Emotional exhaustion mediates the relationship between CCB and facades of conformity*.

## Materials and Methods

### Procedure and Participants

This study utilized a self-reported questionnaire to survey supervisors and employees in the public sector and at a large private bank in Taiwan. We contacted the corporation’s HR managers through personal connections, and they assisted by preparing a list of randomly selected employees. The employee surveys were first distributed to 800 full-time employees who volunteered to participate in this study. All participants were invited to answer the surveys, which measured demographic characteristics, CCB and emotional exhaustion at Time 1 in the workplace. Follow-up data was collected 3 months after the first survey packets were distributed, as previously described (Ng et al., 2010). A total of 702 usable surveys were returned for Time 1 (a return rate of 88%). A total of 702 participants were asked to measure workplace deviance and facades of conformity in the workplace at Time 2. The result at Time 2 was a matched sample of 663 (response rate = 94%). After the exclusion of surveys with unavailable and missing data at Time 2, a total of 655 final surveys were used (480 males, 175 females; average age 30.1). All participants were guaranteed the confidentiality of their responses. The research participants worked in the public sector (42.50%) and at a large private bank (58.50%) in Taiwan.

### Instrument

This study used a Mandarin version of the survey, which was translated from the English version that was developed by native English-speaking researchers. To ensure the content equivalence of the translated version, Chinese speaker, who are proficient in English, were employed to scrutinize all the scales used in this study, following a back-translation procedure during survey construction (Brislin, 1980). The measurement variables for this study included five items, each with a 5-point Likert response scale that ranged from “never” (1) to “always” (5).

### Measurement Variables

#### Compulsory Citizenship Behavior

Drawing on the questionnaire design from Vigoda-Gadot (2007), this section evaluated whether employees engaged in CCB under pressure from their organizations and supervisors at Time 1. The Cronbach’s alpha (α) of this study was 0.83. An example of an item used in this section was: “*There is pressure in the organization that forces me to work overtime without any rewards*.”

#### Emotional Exhaustion

To assess levels of emotional exhaustion in employees, this study utilized a nine-item emotional exhaustion subsection from the Chinese edition of the Maslach Burnout Inventory-General Survey (Lu et al., 2005) at Time 1. For this study, the Cronbach’s alpha (α) was 0.86. The participants assessed their feelings of exhaustion through items such as “*I am tired of my work*.”

#### Workplace Deviance

This study assessed work deviance via an 8-item measure developed by Aquino et al. (1999) to gauge workplace deviance targeted at the organization on a 5-point Likert scale at Time 2. The Cronbach’s alpha (α) of this study was 0.65. The measure included items such as “took undeserved breaks to avoid work” and “intentionally arrived late for work.”

#### Facades of Conformity

Referencing the questionnaire design from Hewlin (2009), this section evaluated whether employees suppressed their personal values, and pretended to embrace organizational values, at Time 2. This six-item scale of this study (α = 0.83) had the same measurement design as the CCB scale. A sample item was: “*I do not pretend to accept the organizational values to achieve my personal purpose*.”

#### Control Variables

The literature suggested that demographic features were likely to affect the results of organizational studies. Therefore, at both Time 1 and Time 2, we incorporated the participant’s sex, age, working hours, and number of children as control variables to minimize errors in the results of the analysis.

## Results

### Measurement Model Analysis

Prior to testing the hypotheses, we conducted a confirmatory factor analysis to estimate the four factor models (CCB, emotional exhaustion, workplace deviance, and facades of conformity). We applied the chi-square, root mean square error of approximation (RMSEA), comparative fit index (CFI), standardized root mean square residual (SRMR), and adjusted goodness of fit index (AGFI) tests as measurement indicators. Table 1 provides the goodness of fit of the models, as follows: chi-square χ*2* (246, N = 655) = 1432.07, *p* < *0.01*; RMSEA = 0.05; CFI = 0.95; SRMR = 0.05; and AGFI = 0.91. All indicators exceeded their thresholds of satisfaction; therefore, the goodness-of-fit of the four-factor models was satisfactory. To verify that the four factor models were optimal, we established three competition models for comparison (Table 1). In addition, according to our factor loading and covariance investigation, all factor loadings in the baseline model were statistically significant (standardized loadings ranged from 0.60 to 0.82), thus supporting convergent validity.

**TABLE 1:** Comparison of measurement models.

Model	Factors	χ^2^	*df*	Δχ^2a^	RMSEA	CFI	SRMR	AGFI
Baseline model	Four factors	1432.07**	246	–	0.05	0.95	0.05	0.91
Model 1	Three factors: Two outcomes were combined into one factor, one mediator and one independent variable	2689.81**	249	1257.74**	0.12	0.92	0.09	0.79
Model 2	Two factors: Two outcomes and one mediator were combined into one factor, and one independent variable	7555.67**	251	6123.60**	0.21	0.83	0.14	0.51
Model 3	One factor: All four factors were combined into one factor.	8878.62**	252	7446.55**	0.23	0.79	0.15	0.47

**p < 0.01.

*a: Baseline model was compared with models 1-3, respectively.*

### Hypothesis Testing

Hypothesis 1 stated that CCB is positively correlated with workplace deviance, which was consistent with the statistical results in Tables 2, 3 (*r* = 0.36, *p* < 0.01). Hypothesis 2 predicted that CCB is positively correlated with facades of conformity, which was consistent with the results in Table 3 (*r* = 0.37, *p* < 0.01). Hypothesis 3 posited that CCB is positively correlated with emotional exhaustion, which also corresponded with the results in Table 3 (*r* = 0.60, *p* < 0.01).

**TABLE 2:** Means, standard deviations, Cronbach’s alpha, and intercorrelations among study.

Variable	*M*	*SD*	1	2	3	4	5	6	7	8	9	10	11
1. Gender	1.27	0.45	(–)										
2. Age	30.1	12.48	−0.11**	(–)									
3. Marriage	1.68	0.47	0.07*	−0.15**	(–)								
4. Number of children	0.39	0.73	–0.06	0.17**	−0.64**	(–)							
5. Education	2.45	0.81	0.18**	0.02	0.01	–0.04	(–)						
6. Work hours	57.03	22.22	–0.03	–0.02	–0.01	0.01	−0.08*	(–)					
7. Seniority	8.10	3.86	−0.34**	0.25**	−0.27**	0.25**	−0.29**	0.06	(–)				
8. CCB	2.73	0.94	–0.04	–0.01	0.03	–0.02	0.04	0.16**	0.08*	(0.83)			
9. Emotional exhaustion	2.74	0.90	0.17	0.01	0.09*	−0.10*	0.03	0.06	0.01	0.60**	(0.86)		
10. Facades of conformity	3.06	0.57	0.06	0.03	0.04	–0.04	–0.01	–0.03	0.06	0.37**	0.29**	(0.83)	
11. Employee deviance	2.09	0.74	–0.01	0.03	0.07	–0.06	0.06	–0.04	−0.13**	0.36**	0.41**	0.28**	(0.65)

*CCB = compulsory citizenship behavior.*

*p < 0.05; **p < 0.01.

**TABLE 3:** Comparison of structural equation models for employee’s psychological strain.

Model	χ^2^	*df*	Δχ^2^	RMSEA	CFI	AGFI
Theoretical Model	6.91**	1	−	0.05	0.99	0.95
Alternative Model 1	21.88**	2	14.97**	0.12	0.97	0.92
Alternative Model 2	47.80**	2	40.89**	0.19	0.92	0.82
Alternative Model 3	68.66**	3	61.75**	0.18	0.90	0.83

**p < 0.01.

*Alternative Model 1: Two full mediations.*

*Alternative Model 2, One partial mediation and one full mediation: A partial mediation model where CCB retains its main effect on workplace deviance and where emotional exhaustion affects workplace deviance.*

*Alternative Model 3: One partial mediation and one full mediation: A partial mediation model where CCB retains its main effect on facades of conformity and where emotional exhaustion affects facades of conformity.*

To ensure the rigor of this study, we constructed four variations of the mediation models for testing. Table 3 presents the model data. Alternative Model 1 was a complete mediation model in which CCB had no major effect on workplace deviance and facades of conformity, but where emotional exhaustion did have an effect on them. Alternative Model 2 was a partial mediation model in which CCB retained its primary effect on workplace deviance, and emotional exhaustion affected workplace deviance as well. Alternative Model 3 was a partial mediation model in which CCB retained its primary effect on facades of conformity, and emotional exhaustion affected facades of conformity as well. The optimal model (χ*2* = 6.91, *df* = 1; RMSEA = 0.05; CFI = 0.99; and AGFI = 0.95) was selected as the theoretical model for this study.

**FIGURE 1:**
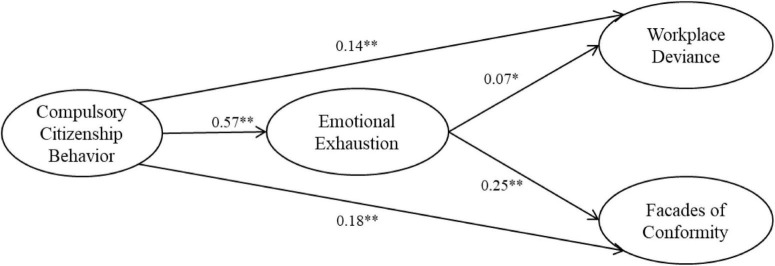
Summary of results. Results are obtained from the structural equation modeling (SEM). *p < 0.05; ^**^p < 0.01.

Figure 1 reveals that CCB had a significant influence on workplace deviance (β = 0.14, *p* < 0.01) and facades of conformity (β = 0.18, *p* < 0.01), supporting Hypotheses 1 and 2, respectively. CCB had a significant and positive effect on emotional exhaustion (β = 0.57, *p* < 0.01), which supports Hypothesis 3. For its analyses of both direct and indirect effects, this study utilized a bootstrapping approach (Preacher and Hayes, 2008) to examine the indirect effect of CCB on workplace deviance and facades of conformity through emotional exhaustion. The findings revealed that CCB had a positive, indirect effect on workplace deviance through emotional exhaustion (*z* = 5.64, *p* < 0.01), since the bootstrapped 99% CI around the indirect effect (0.05, 0.12) did not contain zero, supporting Hypothesis 4. The results also indicated that CCB had a positive, indirect effect on facades of conformity through emotional exhaustion (*z* = 5.22, *p* < 0.01), since the bootstrapped 99% CI around the indirect effect (0.04, 0.15) did not contain zero, supporting Hypothesis 5.

## Discussion

In this study, we investigated the influence of CCB on employee workplace deviance and facades of conformity, and examined how emotional exhaustion mediated the influence and consequences of CCB in the workplace. The results were consistent with Yam et al. (2017), who stated that CCB occurred when employees engaged in citizenship behavior for certain personal interests, which resulted in workplace deviance. Our analysis of the influence of CCB on facades of conformity confirmed that CCB increased the creation of facades of conformity. Moreover, CCB influenced workplace deviance through the mediating effect of emotional exhaustion. In order to obtain rewards or maintain work security, employees may engage in citizenship behavior that is in accordance with the requirements of their organizations or supervisors. However, this excessive consumption of resources (e.g., time and effort) increased employees’ negative emotions, their sense of emotional exhaustion, and their subsequent workplace deviance. Yan et al. (2020) argued that employees were likely to consume more of their personal resources when complying with the additional requirements of their organizations or supervisors. This continual loss of personal resources threatened employees’ well-being, and increased the pressure they experienced. If their additional efforts were not rewarded as expected, employees might exhibit negative behavior as a result of their negative emotions and emotional exhaustion. These empirical results are consistent with those of prior related studies.

### Theoretical Contribution

This study makes three main theoretical contributions to the literature. First, although Liu et al. (2019) discussed the influence of citizenship pressure on work and family conflicts by including CCB as a mediator, prior research has focused on the influence of CCB on employee workplace behavior via their psychological reaction (Liang, 2021). In addition, Hewlin (2009) found that emotional exhaustion mediated the relationship between creating facades of conformity and intentions to leave, because acting in ways incompatible with an individual’s real feelings may generate emotional exhaustion. This study examined how CCB influenced workplace deviance and facades of conformity through emotional exhaustion based on AET. We posited that the emotional exhaustion caused by high-intensity behavior increased employee workplace deviance and facades of conformity; thus, employees exhibited greater workplace deviance and facades of conformity when they engaged in more CCB at the workplace.

Second, we speculated that CCB significantly influenced employees’ creation of facades of conformity. Facade creation refers to false behavior by employees who only pretend to accept organizational values. This reduces employee job satisfaction and increases turnover intention. Accordingly, follow-up studies may examine the influence of citizenship behavior on employees’ psychological and physical health.

Third, we proposed that, because CCB could be considered a type of citizenship behavior, the continual stress that employees experienced, and the additional time and effort they expended, consumed their personal resources and intensified their sense of emotional exhaustion. The results of this study support the concept of emotional exhaustion as a mediator of the influence of CCB on workplace deviance and facades of conformity. Based on the concept of CCB proposed by Vigoda-Gadot (2006), we proved that employees might have negative emotional reactions and behavior, such as workplace deviance and facades of conformity, when external pressure obligates them to spend extra time and effort on citizenship behavior.

### Practical Contribution

This study makes three key practical contributions to the literature. First, although relevant studies have claimed that employees’ OCB could increase organizational performance and competitive advantage, Mo and Shi (2017) indicated that workplace deviance by employees hindered organizational performance. Pressure that organizations impose on employees in response to rapid changes may trigger workplace deviance and inhibit organizational performance.

Second, employees tended to engage in extra-role behavior in order to meet organizational requirements, which might benefit organizational operations in the short term; however, the behavior may eventually stimulate negative emotions in employees, because their external behavior contradicted the values they held and led to negative results such as increased employee turnover intention (Hewlin et al., 2016).

Third, employees were more likely to develop negative emotional reactions due to CCB. Employees were prone to compromising and suppressing their negative emotions in order to protect their personal resources, refraining from expending their resources on emotional outbursts that offended their supervisors. However, this accumulation of negative emotions might lead to emotional exhaustion. Therefore, to reduce the negative influence of emotional exhaustion on employees, management should provide suitable rewards that compensate employees for their resource consumption when additional effort is necessary to increase organizational performance.

Finally, this research may show that followers with deviant behavior and facades of conformity refer to followers experiencing CCB that cause them to conform to the leaders. Followers in organizations who set aside their own values and falsely accept their leaders’ values may not accept involuntary assignments. Therefore, it may suggest that leaders should provide suitable communications when followers experience involuntary assignments.

### Limitations and Future Research

Despite its theoretical and practical contributions, this study has several limitations. First, although the employees completed the questionnaire survey in two stages, we discovered problems related to self-reporting because of single-source bias. Follow-up studies may distribute questionnaires at different times, and from different sources, in order to maximize validity. Second, Wu et al. (2018) suggested that determining methods that encouraged employees to perform extra-role citizenship behavior in the workplace was a crucial challenge for organizations. In addition, Yildiz and Yildiz (2016) argued that an organization’s or supervisor’s interactions with, and responses to, employees might affect the employees’ attitudes and behaviors. Follow-up studies may consider the influence of leadership style on employee CCB. Future studies could also incorporate leader-member exchange theory to examine whether this exchange diminishes or intensifies the influence of leadership style on CCB. Finally, this study did not have an experimental design, and only presented a correlation approach that did not manipulate exploratory variables. Therefore, the exploratory variable in this study might have influenced the dependent variable. In order to capture the organizational processes in the workplace, further research should conduct a laboratory experiment that can limit the subjects’ behavior.

## Data Availability Statement

The original contributions presented in the study are included in the article/supplementary material, further inquiries can be directed to the corresponding author/s.

## Ethics Statement

The studies involving human participants were reviewed and approved by the National Chen Chung University Human Research Ethics Committee. The patients/participants provided their written informed consent to participate in this study.

## Author Contributions

H-LL contributed the idea of the research framework to this study and wrote the whole manuscript. T-KY contributed the questionnaire distribution and the data analysis to this study. C-HW contributed to the response of the reviewers’ comments for this study and revised this study critically for important intellectual content. All authors contributed to the article and approved the submitted version.

## Conflict of Interest

The authors declare that the research was conducted in the absence of any commercial or financial relationships that could be construed as a potential conflict of interest.

## Publisher’s Note

All claims expressed in this article are solely those of the authors and do not necessarily represent those of their affiliated organizations, or those of the publisher, the editors and the reviewers. Any product that may be evaluated in this article, or claim that may be made by its manufacturer, is not guaranteed or endorsed by the publisher.
