# Corrigendum: Compulsory Citizenship Behavior and Its Outcomes: Two Mediation Models

**DOI:** 10.3389/fpsyg.2022.869744

**Published:** 2022-03-01

**Authors:** Huai-Liang Liang, Tsung-Kai Yeh, Chia-Hsuan Wang

**Affiliations:** ^1^College of Management, Dayeh University, Changhua, Taiwan; ^2^Department of Management, Air Force Institute of Technology, Kaohsiung City, Taiwan

**Keywords:** compulsory citizenship behavior, emotional exhaustion, deviant behavior, false behavior, impression management

In the original article, there was an error in the correspondence address provided. Instead of huai-liang@yahoo.com.tw the correct email address should be hliang@mail.dyu.edu.tw.

The authors apologize for this error and state that this does not change the scientific conclusions of the article in any way. The original article has been updated.

## Publisher's Note

All claims expressed in this article are solely those of the authors and do not necessarily represent those of their affiliated organizations, or those of the publisher, the editors and the reviewers. Any product that may be evaluated in this article, or claim that may be made by its manufacturer, is not guaranteed or endorsed by the publisher.

